# Acquired factor VIII deficiency in a nulliparous patient undergoing induction of labor

**DOI:** 10.1515/crpm-2023-0004

**Published:** 2024-03-11

**Authors:** Nawras Zayat, Shirley Huang, Anthony Filipovic, Lorie Bartley, Wissam Akkary

**Affiliations:** 205134 Montefiore Medical Center, Jack D Weiler Hospital, Bronx, NY, USA; 12298 State University of New York, Downstate Health Sciences University, Brooklyn, NY, USA; 24513 Kings County Hospital Center, Brooklyn, NY, USA

**Keywords:** acquired hemophilia A, epidural hematoma, perinatal medicine, subcutaneous hematoma

## Abstract

**Objectives:**

To present a case of acquired factor VIII deficiency in the setting of labor and describe the challenges of its diagnosis and treatment.

**Case presentation:**

A 31-year-old woman was diagnosed with acquired factor VIII deficiency while undergoing induction of labor. Her labor and post operative course were complicated by epidural hematoma formation, prolonged postoperative surgical site bleeding, and subcutaneous hematoma. Management included blood products, human Factor VII, rituximab, and a steroid taper.

**Conclusions:**

Acquired factor VIII deficiency can be challenging to diagnose and should be considered in the differential diagnosis in patients with prolonged bleeding accompanied by a prolonged activated partial thromboplastin time (aPTT).

## Introduction

Acquired hemophilia A (AHA) is a bleeding disorder caused by IgG autoantibodies to clotting factor VIII which may lead to downstream prevention of its binding to factor X, factor Xa and von Willebrand factor, as well as other pathologies [1]. AHA is rare, occurring in 1–2 cases per million per year, predominantly affecting the elderly population with a median age of 68–80 years [2, 3]. AHA is known to be associated with several underlying conditions [4]: malignancy in approximately 10 % of cases, autoimmune disease in another 10 % (such as rheumatoid arthritis [5], systemic lupus erythematosus [6], multiple sclerosis [7]), certain medications (such as antibiotics, antivirals, anticonvulsants) [2, 8], and pregnancy. However, in about 50 % of cases no underlying cause is identified.

AHA presents as soft tissue, mucosal or intramuscular hemorrhage, or at sites related to the underlying conditions – e.g., uterine, cesarean incision and epidural site bleeding in postpartum cases. In more severe instances, gastrointestinal hemorrhage [9], compartment syndrome [10], hemothorax [11] or epidural hematoma as in our case may result. Because of the frequently severe nature of bleeding and delayed recognition, the condition leads to mortality in approximately 22 % of cases, rising to 42 % in the elderly and in malignancy-associated cohorts [3, 12]. AHA is characterized by an isolated activated partial thromboplastin time (aPTT) elevation in setting of mucocutaneous, muscular, or more serious hemorrhage, and previously normal coagulation studies in a patient with no history of bleeding disorder [4]. Mixing studies in which patient’s plasma is mixed with a normal pooled plasma sample may demonstrate immediate correction followed by return to the abnormal range after a 2-h incubation. A significant reduction in factor VIII activity and positive Bethesda assay for inhibitor detection may also be seen [1, 4, 13]. Pharmacologic anticoagulation and lupus anticoagulant must be ruled out as well prior to diagnosis.

Pregnancy-related AHA is typically characterized by onset following a patient’s first pregnancy, commonly in the period one to four months postpartum and up to one year postpartum [14]. The median age at onset is 30 years and symptoms are generally milder with a lower mortality rate – in one systematic review it was 1.7 % [15]. In the same review, relapse after initial remission was reported in 22.2 % of pregnancies. Low inhibitor titers and presentation in the antepartum period are associated with more severe symptoms, longer persistence of inhibitor and development of other autoimmune disorders. The pathophysiology of pregnancy-related AHA is not well understood and it may be the case that its development may occur throughout pregnancy. Because most cases of AHA present in the postpartum period, it was thought that maternal exposure to fetal factor VIII was the predisposing event. However, this is not in line with the low incidence in subsequent pregnancies. Postpartum autoantibodies are more frequently of the IgG1 and IgG3 subclasses and correspond to a Th1-driven response as opposed to a predominantly IgG4 and Th2-driven response in non-pregnancy associated AHA [16, 17]. This suggests a separate underlying mechanism and may explain the different outcomes seen in the two patient groups. Furthermore, transplacental transfer with asymptomatic and symptomatic newborns have been reported [18, 19].

## Case presentation

We report a case of a 31-year-old G2 P0010 African American woman at 40 + 6 weeks’ gestation who presented to the labor and delivery unit with uterine contractions and was admitted for augmentation of labor due to intermittent fetal heart rate category II tracing. Upon physical examination, the patient was noted to be in early labor, but with no progressive cervical changes noted. Her body mass index was 38.7 kg/m^2^. She had no swelling, tenderness, or ecchymosis. She had no history of coagulopathy, thrombosis, trauma, gestational diabetes mellitus, or preeclampsia during prenatal examination. The patient had an uncomplicated elective termination of pregnancy in 2013.

Her pregnancy was complicated by RH negative status for which the patient received Rhogam at 28 weeks’ gestation. In addition, the patient had a past medical history of sickle cell trait.

On hospital day one, she received epidural anesthesia at the L3–L4 level using a 17-gauge spinal needle. After epidural placement, the patient underwent cervical ripening with a cervical balloon that was removed after 5 h due to spontaneous rupture of membranes. The patient was noted to develop intermittent elevated blood pressures with systolic blood pressure ranging from the 140s to the 150s  mmHg and diastolic blood pressures ranging from the 80s to the 90s mmHg. The urine protein-to-creatinine ratio was 0.69. The patient was then noted to have developed acute bleeding from her right antecubital intravenous access site and her epidural injection site. Both epidural catheter and intravenous catheter were removed.

Her white blood cell count at that point was 12.2 k/µL, with 79.8 % neutrophils; her hemoglobin was 11.4 g/L, and platelets were 365 k/µL. Her peripheral blood smear showed 2–3 Schistocytes, mature neutrophils with normal granulation, and mature platelets with normal granulation. Her prothrombin time (PT) was 11.5 and aPTT was 83 s. An aPTT mixing study showed that her aPTTs were 77 and 57 s at 0 and 2 h, respectively, results suggestive of a coagulation factor inhibitor. Factor IX activity was 134 %, factor XI activity was 64 %, and factor VIII activity was <2 %. The Bethesda assay showed a FVIII antibody titer of 72 Bethesda units (BUs). The diagnosis of AHA was confirmed. The patient had a previously normal aPTT on 05/29/2019 (Table 1) making it probable that the diagnosis likely developed during her pregnancy.

**Table 1:** Summary of Laboratory Findings Over Time. This table presents a chronological overview of the patient's laboratory results, including prothrombin time (PT), activated partial thromboplastin time (aPTT), Factor VIII activity, and antibody titer, among others, from the initial hospital admission to post-treatment recovery, highlighting the diagnosis and response to treatment for acquired hemophilia.

Date	05/29/2019	10/28/2020	11/23/2020	12/7/2020	12/21/2020	02/05/2021
PT	12.4 seconds	11.5 seconds				
aPTT	32 seconds	83 seconds	66 seconds	44 seconds	33 seconds	38 seconds
Factor VIII activity		<2 %	1 %	18 %	74 %	54 %
Factor VIII antibody titer		72 BUs	96 BUs		7.0 BUs	0 BUs
Factor IX activity		134 %				
Factor XI activity		64 %				
Hb		11.3 g/dL				
Hct		35.5 %				
Platelet count		365 K/μL				
LDH		197 U/L				
Haptoglobin		227 mg/dL				
Fibrinogen		485 mg/dL				
Uric acid		6.3 mg/dL				
Creatinine		1.09 mg/dL				
ALT		19 U/L				
AST		19 U/L				
Urine protein/creatinine ratio		0.69				
Blood type		O negative				
SARS-CoV 2 RT-PCR		Negative				

aPTT, activated partial thromboplastin time; PT, prothrombin time; BU, Bethesda units; ALT, alanine aminotransferase; AST, aspartate aminotransferase; Hb, hemoglobin; Hct; hematocrit; LDH, lactate dehydrogenase. This Table summarizes the laboratory findings during our patient hospitalization as well as prior to the onset of the illness and after the patient’s discharge.

She was transfused two units of fresh frozen plasma and then underwent cesarean delivery under general anesthesia due to a persistent category II fetal heart rate tracing remote from delivery. The intraoperative estimated blood loss was 800 mL. The patient delivered a live vigorous male neonate, weighing 3,465 g, with APGAR scores of 9 and 9 at 1 and 5 min of life, respectively.

The aPTT remained elevated during her postoperative course even though a total of four units of fresh frozen plasma (two units preoperatively and two units postoperatively) were provided. In addition, the patient received a total of four units pRBCs (one unit intraoperatively and three units postoperatively). During her hospital course, platelets, platelet count, total bilirubin, and haptoglobin remained within normal limits and, as stated before, the peripheral blood smear showed no signs of hemolysis making hemolytic disease and disseminated intravascular coagulopathy (DIC) less likely.

**Figure 1:**
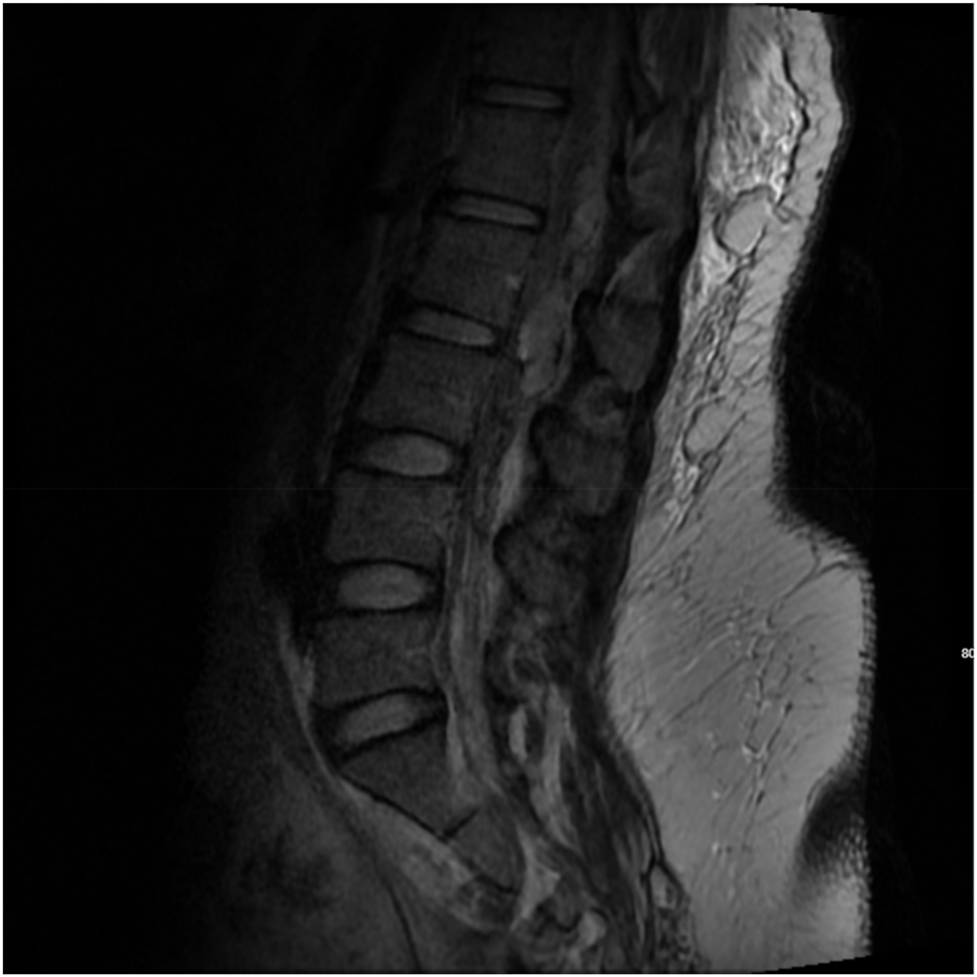
Sagittal MRI of the thoracic and lumbar spine on postoperative day 0. The image reveals a posterior epidural hematoma extending from T12/L1–L4, measuring 1.0 cm anteroposteriorly, 1.1 cm transversely, and 11.5 cm craniocaudally. Significant mass effect on the cauda equina and conus medullaris is evident.

A magnetic resonance imaging (MRI) scan of the spine was performed postoperatively revealing an epidural hematoma within the posterior epidural space extending from the T12/L1 level downwards to the L4 level, and measuring 1.0 cm AP, 1.1 cm transversely, and 11.5 cm craniocaudal with significant mass effect on the cauda equina and conus medullaris (Figure 1). As a result, the patient was transferred to the Neurosurgical Intensive Care Unit for close neurological monitoring. Neurological examination remained reassuring with no focal neurological deficits noted throughout her hospitalization.

**Figure 2:**
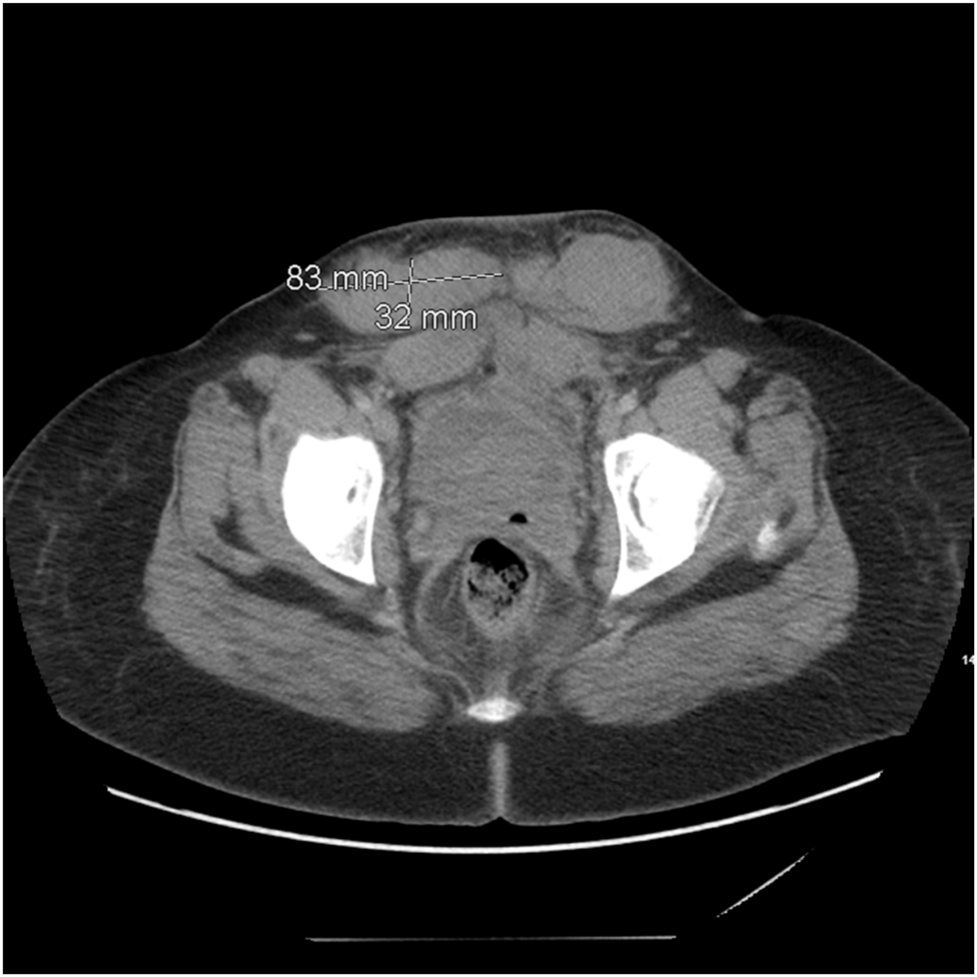
Postoperative day nine CT scan of the abdomen and pelvis following cesarean delivery. Two subcutaneous hematomas visible anterior to the lower rectus musculature at the cesarean skin incision site.

The postoperative course was complicated by an incisional site subcutaneous hematoma (Figure 2), as well as recurrent bleeding from the cesarean incision site. Additionally, the patient developed a left popliteal hematoma measuring ∼2 cm as well as a right medial muscular hematoma affecting the triceps muscle. The treatment regimen for this patient included activated prothrombin complex concentrate (aPCC) aka factor eight inhibitor bypassing activity (FEIBA) (50 U/kg intravenously 3 times daily), human factor VII (Novoseven) 90 μg/kg intravenously, Prednisone 1 mg/kg orally once daily for 4 weeks, and Rituximab 375 mg/m^2^ weekly for total of 4 doses. Of note, human factor VII (Novoseven) was used only as needed in case of bleeding. The decision to use aPCC, human factor VII (Novoseven), or a combination of both depended on several factors, including the severity and location of the bleeding and the clinician preference in consultation with a hemophilia specialist. Due to the risk of thrombotic complications with combined aPCC and human factor VII (Novoseven) treatment, the patient was carefully monitored to ensure optimal dosing and to minimize the risk of thrombotic complications. Regular laboratory monitoring of clotting factor levels, clinical response, and adverse events was performed during treatment. Furthermore, a 10-day course of Aminocaproic acid was used for mild-to-moderate incisional site bleeding that developed on postoperative day eight.

Otherwise, the patient recovered well. She ambulated without assistance. Her postoperative incisional pain was well-controlled with pain medications. Return of normal bladder and bowel function occurred on postoperative day two. A repeat MRI of the spine prior to discharge from the hospital showed interval improvement in the epidural hematoma. The patient was discharged home safely on hospital day 17 with instructions to refrain from contract sports.

Four weeks later, the incisional site had minimal oozing, her aPTT was 66 s, and her hemoglobin was 7.9 g/dL. Factor VIII activity was <1 %, and the factor VIII antibody titer was 96 BU (Table 1).

At the 6-week follow-up visit, bleeding from the incisional site as well as the subcutaneous hematomas had resolved completely, her aPTT was 44 s, and hemoglobin was 10.1 g/dL. Factor VIII activity was 18 % (Table 1).

At the 2-month follow-up, the patient was clinically stable with no complaints. She reported no recent easy bruising or bleeding. aPTT normalized 33 s, hemoglobin 11.9 g/dL. Factor VIII activity normalized (74 %) and the factor VIII antibody titer was 7.9 BU (Table 1). Prednisone was successfully tapered over a period of four weeks was started after aPTT normalized and factor VIII inhibitor decreased significantly. The patient underwent 6-month follow-up visits and showed no recurrence. Factor VIII activity as well as APTT continued to be normal.

## Discussion

We have reported a rare case of acquired factor VIII deficiency complicated by epidural hematoma formation, prolonged postoperative surgical site bleeding, and subcutaneous hematoma. Because the bleeding symptoms in our patient presented specifically within the intrapartum period, we conducted a literature review to search for other reported cases presenting in a similar time frame. Most case reports described disease with onset in the period within one week [10, 20], [21], [22], [23], [24], [25], [26], [27], one month [14, 28] or up to eight months [14, 15, 22, 29] following delivery. Often case reports or series did not specify the timing of symptoms in relation to delivery, including cases used in large patient registries from Taiwan, Australia and Italy [30], [31], [32], [33]. However, our literature review found three cases of antepartum and one case of intrapartum AHA. The only intrapartum case encountered [34] was that of a 26-year-old G3P2 whose initial presentation was bleeding from the epidural catheter site, similar to our case. That patient also developed an epidural site hematoma, hematuria and, after diagnosis and treatment during the same hospital stay, returned twice for vaginal bleeding. Her antibody eradication treatment was ongoing at the time of the case report publication. In the first antepartum case [35], a 28-year-old woman at 22 weeks of gestation developed uncontrollable intravenous access site bleeding during treatment for a relapse of generalized pustular psoriasis. That is particularly interesting because of the presence of two common predisposing factors to AHA. In the second antepartum case [36], a 31-year-old woman undergoing her first IVF cycle developed severe ovarian hyperstimulation syndrome, necessitating intensive care. Initially admitted for ovarian hyperstimulation syndrome three days post-embryo transfer, her condition initially stabilized. However, on hospital day 29, she presented with new-onset hematuria and ecchymosis on her lower extremity, symptoms not previously observed. These developments led to the diagnosis of acquired hemophilia, confirmed by the detection of a clotting inhibitor against factor VIII. A third case of antepartum AHA was disclosed in an analysis of the EACH2 registry [37], however no further information was specified about the patient. The two antepartum patients for whom information is available successfully accomplished antibody eradication. While the patient in the intrapartum case was para 2, the first two antepartum cases were primigravid, and in the EACH2 registry 73.8 % of pregnancy-related cases were in primigravid women. This is in line with the known association of the condition with first pregnancies.

The purpose of this case report is to raise awareness among health-care workers about the presentation, diagnosis, and early treatment of pregnancy-related AHA. AHA mainly manifests as hemorrhages in the skin, mucous membranes, and muscles. In our case, the patient presented with intrapartum prolonged IV venipuncture and epidural injection sites bleeding with abnormal coagulation function, which is rarely reported in other cases. Therefore, the diagnosis of pregnancy-related acquired hemophilia should be considered in women with abnormal coagulation function accompanied by prolonged bleeding from intravenous access and epidural injection sites. In our case, the patient had prolonged aPTT, low FVIII, and the presence of FVIII inhibitors.

The two goals of AHA treatment are management of the acute hemorrhage and to suppress the formation of the inhibitory autoantibodies. Treatment should aim to balance the need to quickly eliminate the inhibitory antibodies and minimize the side effects of immunosuppressive therapy [11]. Multiple hemostatic agents are available that either overwhelm autoantibodies (by replacing inhibited clotting factor) or that bypass the inhibited step in the coagulation cascade [2, 38]. Replacement therapies include human factor VIII concentrate and desmopressin. Desmopressin is thought to transiently raise factor VIII levels [39]. An updated set of guidelines based on data from multiple AHA patient registries [19] recommend use of factor VIII augmentation/replacement only if bypassing agents are unavailable or ineffective, and if inhibitor titers are low. The use of tranexamic acid, an antifibrinolytic, is controversial and thought to be associated with risk of thrombosis [40]. Porcine factor VIII, originally considered a bypassing agent, is widely used today as an important first-line therapy [41]. Other first-line therapies include recombinant factor VIIa and aPCC products (such as factor eight inhibitor bypassing activity, or FEIBA) [42], [43], [44]. An in-depth study of outcomes of patients in a European AHA patient registry, the EACH2 registry, showed that delay in time to treatment was the only factor that significantly differed between treatment responders and non-responders [45]. It also showed superiority of bypassing agents over human factor VIII concentrate and desmopressin. In addition to the above, packed red blood cell transfusions and avoidance of invasive procedures when possible are practiced.

Eradication of the inhibitor should be initiated with corticosteroids as soon as AHA is diagnosed, even while hemostatic therapy is taking place. Aggressive suppressive treatment with steroids (most commonly prednisone) with or without cyclophosphamide was recommended due to high bleed-related mortality was previously recommended [19]. However, a European prospective observational study showed that this approach resulted in high treatment-related mortality [40], so the current favored approach is to individualize treatment based on severity of disease and inhibitor titers. Cyclophosphamide, vincristine or Rituximab (a monoclonal antibody targeting the B-cell-specific CD20 protein) are now used as add-on therapy in cases of initial steroid therapy failure [38, 43, 46]. Furthermore, intravenous immunoglobulin and immunoadsorption are available for particularly severe cases. The underlying condition, if present, also governs eradication therapy, with less aggressive therapy reserved for postpartum cases as opposed to those associated with autoimmune conditions or malignancy [2]. Our patient responded to treatment with corticosteroids and rituximab and went into remission.

**Figure 3:**
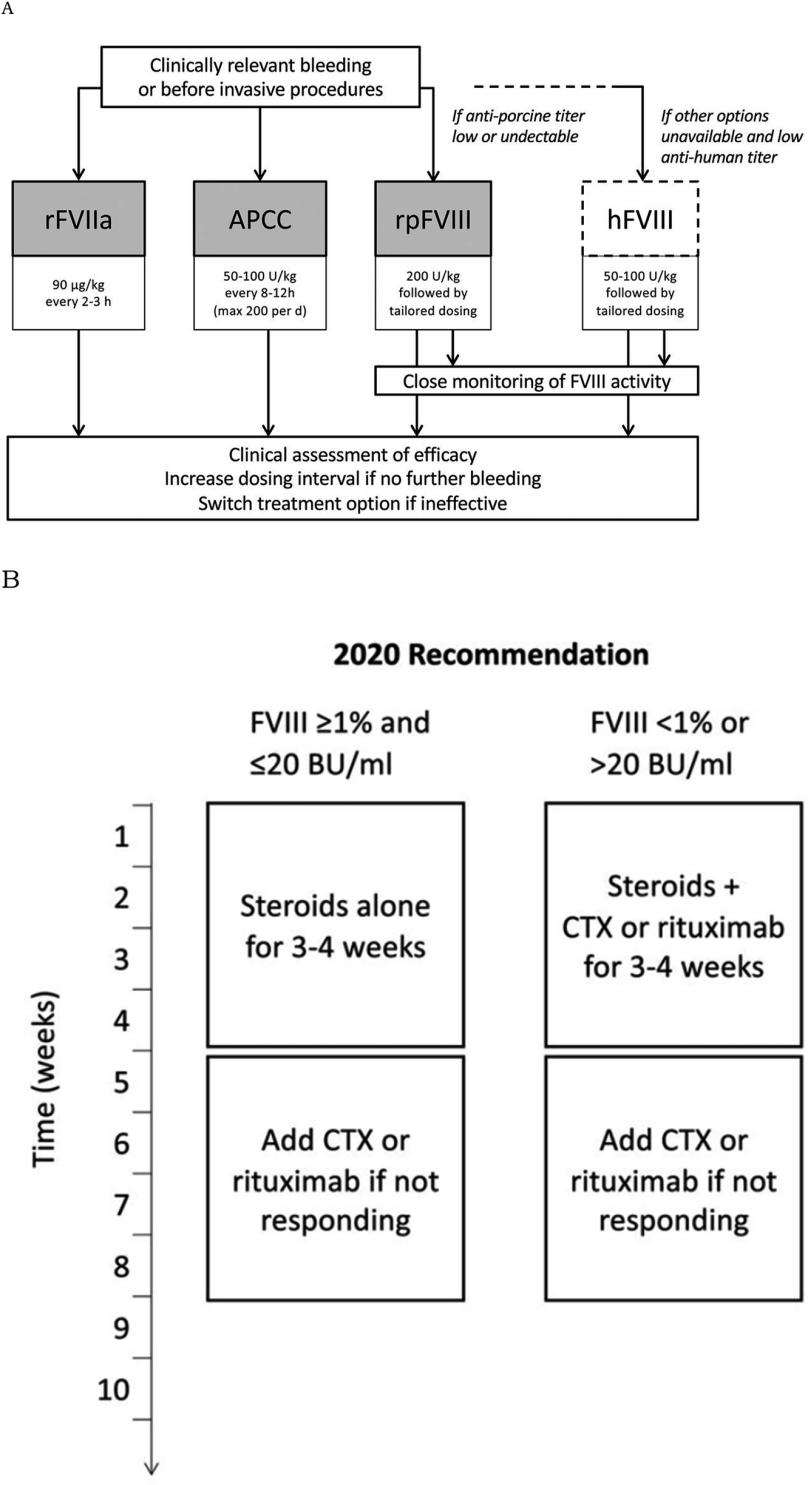
Comprehensive treatment approach for acquired hemophilia A. This figure illustrates the dual strategy in managing AHA, highlighting both hemostatic treatments to address acute bleeding episodes and immunosuppressive therapies aimed at eradicating the underlying autoantibodies. (A) Treatment guidelines for hemostasis in patients with AHA as per International Guidelines (rFVIIa, recombinant activated factor VII; aPCC, activated prothrombin complex concentrate; rpFVIII, recombinant porcine factor VIII; hFVIII, human (plasma-derived or recombinant) factor VIII; h, hour; d, day). (B) Immunosuppressive recommendations for patients with AHA as per International Guidelines (FVIII, factor VIII activity; BU, Bethseda unit; CTX, cyclophosphamide).

Figure 3A and B illustrates the 2020 International Recommendations regarding hemostatic and immunosuppressive treatment of AHA patients [13].

## Conclusions

Pregnancy-related acquired factor VIII deficiency should be considered in pregnant patients with prolonged bleeding from IV venipuncture and epidural injection sites accompanied by prolongation of aPTT. Prolonged PTT that fails to correct by adding fresh plasma suggests the presence of the inhibitor and should trigger further workup. If the diagnosis of autoimmune acquired factor VIII deficiency is confirmed, treatment should be initiated promptly to suppress the formation of the inhibitory autoantibodies. Delayment of both diagnosis and treatment may lead to many serious complications, especially in patients who require procedures such as epidurals and c-sections. One such example is expansion of an epidural hematoma that can cause clinically significant spinal cord compression, further leading to neurological impairment. Furthermore, the use of corticosteroids and Rituximab in our patient proved to be safe and efficacious for the treatment of acquired factor VIII deficiency, leading to suppression of the formation of the autoantibodies and remission of the disease.
